# Impacts of Rotation-Fallow Practices on Bacterial Community Structure in Paddy Fields

**DOI:** 10.1128/spectrum.00227-22

**Published:** 2022-07-27

**Authors:** Na Li, Xinmei Li, Shujuan Li, Shujian Guo, Ziwei Wan, Guoqin Huang, Huifang Xu

**Affiliations:** a Research Center on Ecological Sciences, Jiangxi Agricultural University, Nanchang, China; b Key Laboratory of Crop Physiology, Ecology and Genetic Breeding, Ministry of Education/Jiangxi Province, Jiangxi Agricultural University, Nanchang, China; University of California, Davis

**Keywords:** crop rotation-fallow, bacteria, community structure, paddy field

## Abstract

Soil nutrients and microbial community play a central role in determining crop productivity in agroecosystems. However, the relationship between microbial community structure and soil nutrients in various crop rotation-fallow systems remains unclear. Thus, we designed a 3-year crop rotation-fallow field with five cropping systems (one continuous cropping, three rotational cropping, and one fallow system). We conducted a comprehensive analysis by evaluating crop yield, soil physicochemical properties, and overall bacteria composition. Our results showed that rotation-fallow treatments markedly influenced the crop yield and soil physicochemical properties. *Proteobacteria*, *Acidobacteriota*, and *Chloroflexi* were the dominant phyla in all rotation-fallow treatments. pH, available-phosphorus, total nitrogen, and soil organic matter had considerable effects on the soil bacterial community structure in 2019; however, only available-phosphorus had an impact on soil bacterial community in each treatment in 2020. In summary, with the increase of tillage years, different rotational fallow systems can increase paddy yield by promoting soil nutrient uptake and increasing the relative abundances of bacteria in paddy fields.

**IMPORTANCE** Soil nutrients and microbial community play a central role in determining crop productivity. Therefore, elucidating the microbial mechanisms associated with different cropping systems is indispensable for understanding the sustainability of agroecosystem. In the present study, we designed a 3-year field rotation experiment using five cropping systems, including one continuous cropping, three rotational cropping, and one fallow system, to indagate the outcomes of soil microbial community structures in the different tillage systems. Our results showed that the different rotational fallow systems had positive impacts on crop yield, soil physicochemical properties, and bacterial community structure and that available phosphorus might be a key determinant for the limited bacterial community structure in various rotation-fallow systems following a 3-year field experiment. This study suggests that crop rotation-fallow systems play critical roles in improving bacterial community structure.

## INTRODUCTION

Rice is an important cereal, which serves as a staple food for more than half of the world’s population (1). The crop rotation-fallow system, rather than the traditional continuous cropping model, should be adopted for sustainable rice production, which incorporates the cultivation of food, cash, and fertilizer crops to generate higher economic (2, 3) and soil health benefits (4) in China. The rotation-fallow system not only decreases the need for fertilizer application, but also promotes crop diversities (5–7). However, numerous studies have shown that although a continuous/monoculture system of cropping can improve crop yield in the short term, it has various long-term pitfalls, such as root toxicity by organic and phenolic acid accumulation (8), soil nutrient depletion (9), pathogenic microbial infestation (10), and biodiversity reduction (11), thereby ultimately affecting crop production and sustainability of the soil environment (12). Therefore, understanding how crop rotation-fallow systems affect soil properties, microbial community composition, and crop field is indispensable for food security and environmental health in the future.

Increasing the diversity of crop rotation is the main approach to overcoming the negative influence of monocultures in intensive cropping systems (13). Rotation cultivation with double- or triple-crop systems has been practiced for years as an effective form of land management in southern China (14). In a paddy-upland rotation system, paddy rice (Oryza sativa) is generally cultivated in the summer followed by upland crops in the winter, such as milk vetch (*Astragalus sinicus L.*), oilseed rape (*Brassica napus L.*), soybean [Glycine max (*L.*) *Merrill*], maize (Zea mays *L.*), sugarcane (*Saccharum officinarum L.*), and sweet potato [*lpomoea batatas* (*L.*) *Lam.*] (3, 5–9). Previous studies have mainly focused on elucidating the influence mechanism of different crop rotation systems on the soil microbial community’s biomass, structure, and activity (15). Specifically, Xuan et al. (16) and Linh et al. (17) reported that the soil bacterial community structure was significantly different in rotational cropping treatment, compared with single-cropping rice. Furthermore, Zhang et al. (18) reported that soybean-maize rotation played a marked role in maintaining the stability of the soil microbial community. Meanwhile, the fallow system has been identified as an effective approach to prevent soil acidification and secondary latent soil fertilization (19, 20). Wang et al. (21) reported that fallow systems significantly increased microbial diversity in agricultural soils. However, the intrinsic association of microbial community structure with soil environmental factors under different rotation-fallow systems has not been fully elucidated.

In the present study, five cropping rotation-fallow systems were performed in a field from 2018 to 2020. Soil samples were collected for comparing the effects of the different cropping treatments on soil bacterial community structure and to elucidate the intrinsic association between the bacterial community structure and soil environmental factors under the five rotation-fallow systems. The objective of this study was to demonstrate that the increase of tillage years could invariably improve soil fertility and relative bacterial community diversity in a paddy field by adopting various rotation-fallow systems, which will ultimately increase crop yield.

## RESULTS

### Crop yield.

**FIG 1:**
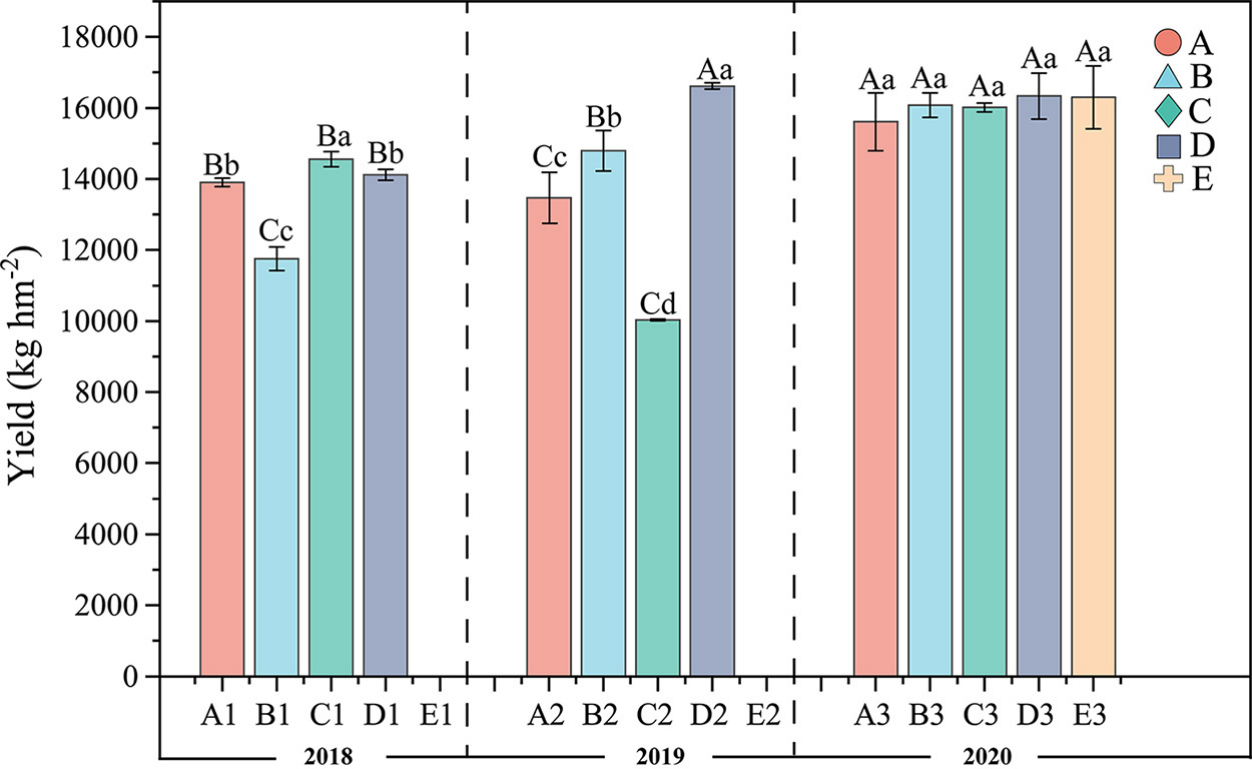
Crop yield of different rotational cropping systems. Treatments: A: milk vetch - double cropping rice → milk vetch - double cropping rice → milk vetch - double cropping rice; B: milk vetch - spring soybeans - autumn soybeans → rape - sugarcane ‖ spring soybeans → milk vetch - double cropping rice; C: milk vetch - early rice - maize ‖ sweet potato → milk vetch - spring soybeans - autumn soybeans → milk vetch - double cropping rice; D: rape - sugarcane ‖ spring soybeans → milk vetch - early rice - maize ‖ sweet potato → milk vetch - double cropping rice; E: fallow → fallow → milk vetch - double cropping rice. “-”: multiple cropping; “‖”: intercropping.

The results revealed that different rotation tillage systems had a significant (*P < *0.05) influence on the crop yield in 2018 and 2019, and the crop yields of all treatments were higher than that of the first 2 years (Fig. 1). Both treatments B and C of the respective 2018 and 2019 crop rotation had the least yields of “milk vetch - spring soybean - autumn soybean.” However, both treatments C and D of the respective 2018 and 2019 crop rotation had the highest crop yields of “milk vetch - early rice-maize ‖ sweet potato.” From the overall yield results of the 3-year experiment, the crop rotation-fallow systems all improved paddy yields, with more stable yields in treatment D. Following 2 years of fallow crop rotation, we found that the crop yields under the different rotation-fallow treatments in 2020 were higher than those of 2018 and 2019.

### Soil physicochemical properties.

Different rotation-fallow cropping systems had significant (*P < *0.05) effects on soil physicochemical properties in 2018, 2019, and 2020 (Table 1). In 2018, there was no significant effect on soil physicochemical properties; however, there was a significant (*P < *0.05) difference between the crop rotation-fallow systems in 2019 and 2020. Among them, the soil physicochemical properties pH, TN, and SOM showed a constant decreasing trend from 2018 to 2020; however, alkali-hydrolyzable nitrogen (AN), available phosphorus (AP), and available potassium (AK) were higher in 2019 than in 2018 and 2020.

**TABLE 1:** Soil physicochemical properties among different rotational cropping systems

Treatments^*a*^	pH^*b*^	AN (mg kg^−1^)	AP (mg kg^−1^)	AK (mg kg^−1^)	TN (g kg^−1^)	SOM (g kg^−1^)
2018						
A1	5.36 ± 0.04^*c*^A^*d*^a^*e*^	163.88 ± 3.22 Aa	39.72 ± 7.34 Aa	110.7 ± 14.92 Aa	2.06 ± 0.38 Aa	46.92 ± 3.08 Aa
B1	5.02 ± 0.07 Aa	163.44 ± 12.77 Aa	54.07 ± 16.04 Aa	111.78 ± 49.56 Ba	2.06 ± 0.17 Aa	41.79 ± 1.91 Aa
C1	5.28 ± 0.32 Aa	156.44 ± 2.47 Ba	60.67 ± 10.70 Aa	145.94 ± 57.24 Ba	2.01 ± 0.10 Aa	40.75 ± 183 Aa
D1	5.23 ± 0.19 Aa	161.44 ± 15.91 Ba	46.63 ± 13.39 Ba	119.45 ± 58.46 Ba	2.00 ± 0.21 Aa	47.24 ± 2.84 Aa
E1	5.08 ± 0.08 Aa	160.06 ± 12.14 Ba	45.95 ± 19.56 Aa	168.39 ± 18.13 Ba	2.06 ± 0.12 Aa	45.15 ± 4.31 Aa
2019						
A2	5.28 ± 0.08 Aa	170.81 ± 3.50 Ac	42.57 ± 16.18 Ac	59.00 ± 4.36 Bc	2.00 ± 0.15 Aa	40.19 ± 2.30 Bb
B2	4.34 ± 0.17 Bc	191.31 ± 10.50 Ab	74.68 ± 1.70 Aab	179.00 ± 14.80 Aab	1.82 ± 0.03 Bb	36.05 ± 0.23 Bc
C2	4.76 ± 0.09 Bb	176.98 ± 0.58 Abc	63.80 ± 5.28 Aab	164.00 ± 27.19 Ab	1.84 ± 0.46 ABb	40.90 ± 1.22 Ab
D2	4.51 ± 0.09 Bc	225.14 ± 14.57 Aa	77.21 ± 0.95 Aa	182.00 ± 3.00 Aab	1.85 ± 0.02 Ab	47.74 ± 2.67 Ba
E2	4.81 ± 0.13 Bb	176.81 ± 3.77 Abc	61.48 ± 5.52 Ab	200.00 ± 11.27 Aa	1.88 ± 0.03 Ab	38.35 ± 0.58 Bbc
2020						
A3	4.40 ± 0.10 Ba	172.67 ± 12.29 Aa	48.69 ± 4.15 Ac	26.33 ± 3.06 Cab	2.01 ± 0.18 Aa	34.45 ± 2.09 Ca
B3	4.43 ± 0.12 Ba	168.00 ± 9.26 Aa	73.51 ± 16.90 Aa	19.67 ± 4.73 Cb	1.87 ± 0.10 Aab	35.28 ± 1.97 Ba
C3	4.21 ± 0.57 Cb	147.00 ± 7.00 Bc	65.98 ± 1.74 Aab	34.33 ± 4.04 Ca	1.77 ± 0.11 Bab	36.83 ± 2.17 Ba
D3	4.19 ± 0.47 Bb	156.33 ± 8.81 Bab	56.56 ± 2.79 Bbc	25.00 ± 7.00 Cb	1.81 ± 0.25 Ab	31.83 ± 0.93 Ba
E3	4.17 ± 0.08 Cb	154.00 ± 6.06 Bbc	55.57 ± 8.93 Abc	28.00 ± 1.73 Cab	1.83 ± 0.05 Bab	38.09 ± 3.27 Ba

a Treatments: A: milk vetch - double cropping rice → milk vetch - double cropping rice → milk vetch - double cropping rice; B: milk vetch - spring soybeans - autumn soybeans → rape - sugar-cane ‖ spring soybeans → milk vetch - double cropping rice; C: milk vetch - early rice - maize ‖ sweet potato → milk vetch - spring soybeans - autumn soybeans → milk vetch - double cropping rice; D: rape - sugarcane ‖ spring soybeans → milk vetch - early rice - maize ‖ sweet potato → milk vetch - double cropping rice; E: fallow → fallow → milk vetch - double cropping rice.

b Soil physicochemical properties: TN, total nitrogen; SOM, soil organic matter; AN, available nitrogen; AP, available phosphorus; AK, available potassium.

c Average ± standard deviation (*n* = 3).

d Different letters (A, B, C) represent significant differences in different years at *P *< 0.05.

e Different letters (a, b, c) represent significant differences among different crop rotation-fallow in 1 year at *P *< 0.05.

### Diversity of soil bacterial community.

Different crop rotation-fallow systems had significant (*P < *0.05) effects on bacterial α-diversity index in paddy soils (Table 2). The α-diversity of soil bacteria was significantly (*P < *0.05) lower in 2018 and 2019 than in 2020, except for the Simpson. We also found that the bacterial α-diversity index of treatment A in 2018 was significantly higher than that of the other treatments, with higher range values of Chao1, abundance-based coverage estimator (ACE), and Shannon indices as 6.24% to 17.10%, 7.37% to 17.72%, and 2.96% to 22.91%, respectively. However, the bacterial α-diversity index of treatment D in 2019 was significantly higher compared to other treatments, with higher range values of Chao1, ACE, and Shannon indices as 1.70% to 279.56%, 1.60% to 9.00%, and 1.41% to 13.83%, respectively. Notably, there was no significant (*P > *0.05) difference in bacterial α-diversity index among the treatments in 2020. The results showed that different crop rotation-fallow systems had more significant effects on the bacterial α-diversity index of paddy fields in an increasing tillage year-dependent manner.

**TABLE 2:** Diversity of bacteria among different rotational cropping systems

Treatments^*a*^	Simpson^*b*^	Chao1	ACE	Shannon
2018				
A1	0.0035 ± 0.0005^*c*^ A^*d*^a^*e*^	3,388.11 ± 26.26 Ba	3,357.93 ± 35.63 Ba	6.60 ± 0.06 Ca
B1	0.0056 ± 0.0016 Ba	3,189.12 ± 238.05 Bab	3,127.49 ± 225.98 Bab	6.33 ± 0.18 Ba
C1	0.0584 ± 0.0874 Aa	2,893.41 ± 125.37 Bb	2,852.55 ± 105.85 Bb	5.37 ± 1.15 Ab
D1	0.0056 ± 0.0019 Aa	3,167.66 ± 228.99 Bab	3,094.24 ± 240.11 Bab	6.41 ± 0.20 Ba
E1	0.0049 ± 0.0040 Aa	3,118.56 ± 230.33 Bab	3,094.51 ± 191.93 Cab	6.41 ± 0.11 Ba
2019				
A2	0.0104 ± 0.0102 Ab	3,250.83 ± 237.28 Ba	3,316.17 ± 227.62 Ba	6.42 ± 0.30 ABa
B2	0.0324 ± 0.0161 Aa	3,086.99 ± 91.47 Ba	3,147.84 ± 98.55 Ba	5.64 ± 0.18 Cb
C2	0.0054 ± 0.0014 Ab	3,255.00 ± 162.98 Ba	3,267.74 ± 136.45 Ba	6.35 ± 0.22 ABa
D2	0.0045 ± 0.0001 ABb	3,366.55 ± 132.95 Ba	3,430.91 ± 152.01 Ba	6.51 ± 0.04 Ba
E2	0.0045 ± 0.0009 Ab	3,310.16 ± 146.7 Ba	3,377.18 ± 127.79 Ba	6.51 ± 0.08 Ba
2020				
A3	0.0023 ± 0.0001 Aa	4,770.36 ± 123.35 Aa	4,827.81 ± 95.28 Aa	7.00 ± 0.03 Aa
B3	0.0030 ± 0.0006 Ba	4,660.81 ± 330.56 Aa	4,651.48 ± 399.34 Aa	6.85 ± 0.15 Aa
C3	0.0028 ± 0.0004 Aa	4,488.92 ± 321.16 Aa	4,547.93 ± 390.18 Aa	6.85 ± 0.13 Ba
D3	0.0030 ± 0.0003 Ba	4,573.31 ± 162.01 Aa	4,639.06 ± 138.28 Aa	6.93 ± 0.03 Aa
E3	0.0027 ± 0.0004 Ba	4,749.62 ± 86.62 Aa	4,745.51 ± 44.76 Aa	6.90 ± 0.06 Aa

a Treatments: A: milk vetch - double cropping rice → milk vetch - double cropping rice → milk vetch - double cropping rice; B: milk vetch - spring soybeans - autumn soybeans → rape - sugar-cane ‖ spring soybeans → milk vetch - double cropping rice; C: milk vetch - early rice - maize ‖ sweet potato → milk vetch - spring soybeans - autumn soybeans → milk vetch - double cropping rice; D: rape - sugarcane ‖ spring soybeans → milk vetch - early rice - maize ‖ sweet potato → milk vetch - double cropping rice; E: fallow → fallow → milk vetch - double cropping rice. “-”: multiple cropping; “‖”: intercropping.

b Soil bacterial diversity index: Simpson; Chao1; ACE; Shannon.

c Average ± standard deviation (*n* = 3).

d Different letters (A, B, C) represent significant differences in different years at *P *< 0.05.

e Different letters (a, b, c) represent significant differences among different crop rotation-fallow in 1 year at *P *< 0.05.

### Composition of soil bacterial community structure.

**FIG 2:**
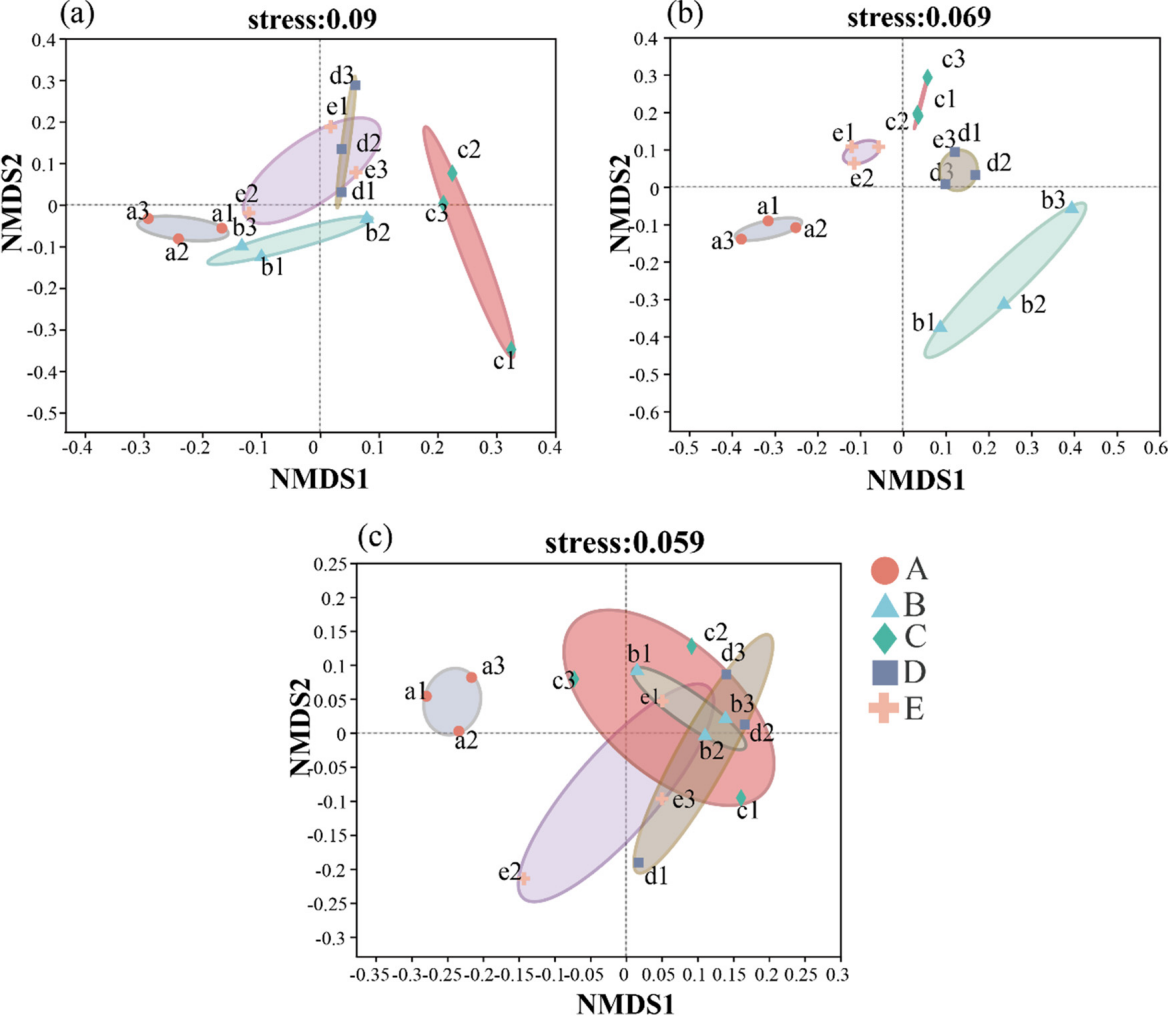
NMDS analysis of different rotational cropping systems. NMDS on OTU level analysis of five different planting systems in 2018 (a); NMDS on OTU level analysis of five different planting systems in 2019 (b); NMDS on OTU level analysis of five different planting systems in 2020 (c). Treatments: A: milk vetch - double cropping rice → milk vetch - double cropping rice → milk vetch - double cropping rice; B: milk vetch - spring soybeans - autumn soybeans → rape - sugarcane ‖ spring soybeans → milk vetch - double cropping rice; C: milk vetch - early rice - maize ‖ sweet potato → milk vetch - spring soybeans - autumn soybeans → milk vetch - double cropping rice; D: rape - sugarcane ‖ spring soybeans → milk vetch - early rice - maize ‖ sweet potato → milk vetch - double cropping rice; E: fallow → fallow → milk vetch - double cropping rice.

Soil bacterial community structure in paddy fields had varying responses to the different crop rotation-fallow systems (Fig. 2) (Table S2). Aside from the area overlap between treatments D and E in 2018, there was no overlap between the other treatments in 2018 and 2019. Furthermore, analysis of similarities (ANOSIM) results (Table S4) confirmed that there were significant (*P < *0.01) differences in the soil bacterial community structure among the five-crop rotation-fallow systems between 2018 and 2019. However, there was a large overlap in the soil bacterial community structure of all treatments “BCDE” except treatment A in 2020, indicating a significant difference in the soil bacterial community structure between all the treatments. In addition, ANOSIM results showed that there were significant (*P < *0.01) differences in soil bacterial community structure in 2020. The overall results showed that different crop rotation-fallow systems had significant effects on the soil bacterial community structure in paddy fields, and this difference is directly proportional to increase in fallow years; however, the significant difference of soil bacterial community structure decreased following the sowing of early rice.

### Phylum-level community structure of soil bacteria.

**FIG 3:**
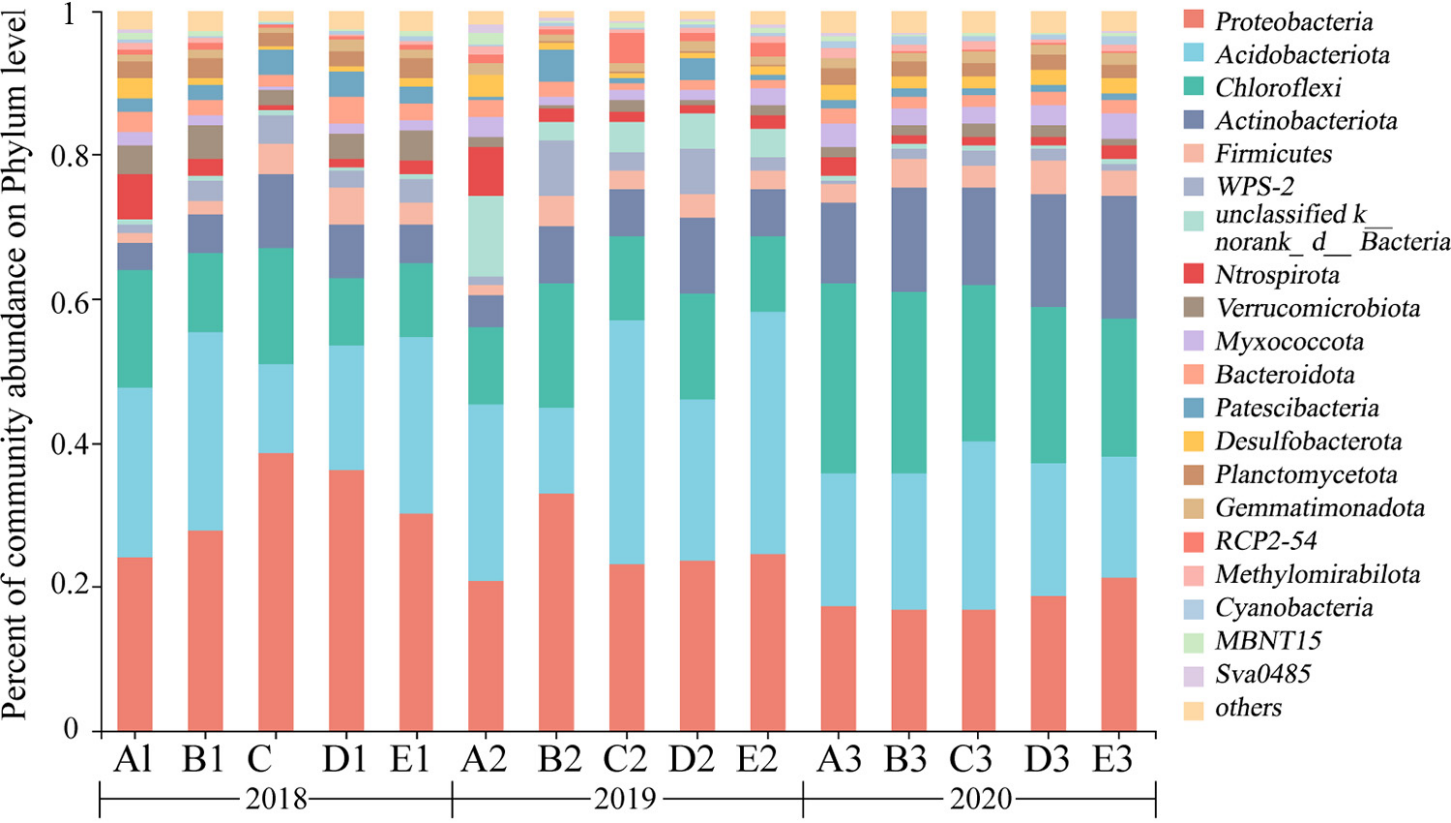
Phylum compositions of soil bacteria in different rotational cropping systems. Treatments: A: milk vetch - double cropping rice → milk vetch - double cropping rice → milk vetch - double cropping rice; B: milk vetch - spring soybeans - autumn soybeans → rape - sugarcane ‖ spring soybeans → milk vetch - double cropping rice; C: milk vetch - early rice - maize ‖ sweet potato → milk vetch - spring soybeans - autumn soybeans → milk vetch - double cropping rice; D: rape - sugarcane ‖ spring soybeans → milk vetch - early rice - maize ‖ sweet potato → milk vetch - double cropping rice; E: fallow → fallow → milk vetch - double cropping rice. A1, B1, C1, D1, and E1 treatments were planted in 2018; A2, B2, C2, D2, and E2 treatments were planted in 2019; A3, B3, C3, D3, and E3 treatments were planted in 2020.

Bacterial phyla abundances in paddy fields under the different rotation tillage systems varied significantly (*P < *0.05) (Fig. 3) (Table S3). Our results showed that *Proteobacteria*, *Acidobacteriota*, and *Chloroflexi* were the dominant phyla in all treatments, and their respective relative abundances in 2018, 2019, and 2020 were as follows: *Proteobacteria*: 25.79% to 40.22%, 21.09% to 35.95%, and 16.31% to 21.50%; *Acidobacteriota*: 12.15% to 29.11%, 10.48% to 35.34%, and 16.42% to 23.17%; *Chloroflexi*: 9.03% to 17.28%, 9.81% to 16.99%, and 18.94% to 26.52%. Aside from *Proteobacteria*, there was a significant (*P < *0.05) effect on the abundance of all other dominant phyla in 2018. However, in 2019, there was a significant (*P < *0.05) effect on the abundance of all dominant phyla except *Chloroflexi*. In contrast to *Proteobacteria* and *Acidobacteriota*, there was no significant (*P > *0.05) effect on the relative abundance of *Chloroflexi* in 2020 (Table S3). Unlike the *Chloroflexi* phylum, which had no significant effect (*P > *0.05), the abundance of *Proteobacteria* and *Acidobacteriota* was significantly lower in 2020 than in 2018 and 2019. We also found that among the bacterial phyla community, bacilli were predominant, including *Proteobacteria*, *Acidobacteriota*, *Firmicutes*, and *Bacteroidota*.

### Correlation between bacterial community structure and soil properties.

**FIG 4:**
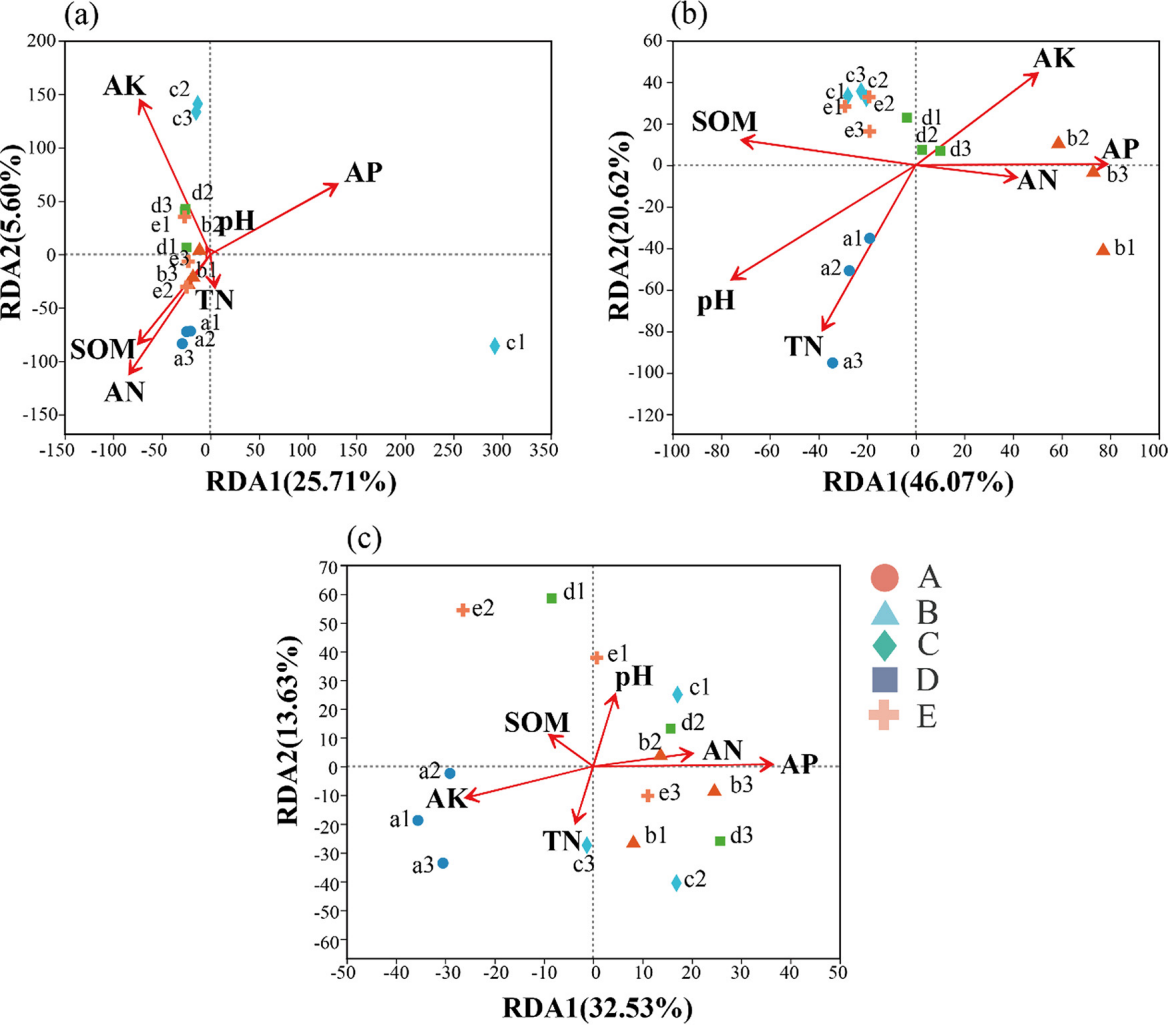
Correlation between bacterial community structure and soil properties in 2018 (a), 2019 (b), 2020 (c). TN, total nitrogen; SOM, soil organic matter; AN, available nitrogen; AP, available phosphorus; AK, available potassium. Treatments: A: milk vetch - double cropping rice → milk vetch - double cropping rice → milk vetch - double cropping rice; B: milk vetch - spring soybeans - autumn soybeans → rape - sugarcane ‖ spring soybeans → milk vetch - double cropping rice; C: milk vetch - early rice - maize ‖ sweet potato → milk vetch - spring soybeans - autumn soybeans → milk vetch - double cropping rice; D: rape - sugarcane ‖ spring soybeans → milk vetch - early rice - maize ‖ sweet potato → milk vetch - double cropping rice; E: fallow → fallow → milk vetch - double cropping rice.

We found that the influencing factors on the soil bacterial community structure varied under different rotation tillage systems in 2018, 2019, and 2020 (Fig. 4; Table 3). Although AK, AP, and AN all had a greater effect on soil bacterial community structure in 2018 than other physicochemical properties, these factors did not achieve the significant (*P > *0.05) level. However, in 2019, pH, AP, TN, and SOM had significant (*P < *0.05) effects on the soil bacterial community structure. Following early rice cultivation in 2020, only AP had a significant (*P < *0.05) effect on soil bacterial community structure in each treatment.

**TABLE 3:** Correlation between bacterial community structure and soil properties in 2018, 2019, and 2020

Soil physicochemical properties^*a*^	*R*	*P* value
2018		
pH	0.0002	0.995
AN	0.2838	0.155
AP	0.2703	0.117
AK	0.2402	0.222
TN	0.0094	0.934
SOM	0.1781	0.349
2019		
pH	0.7629	0.001^*b*^
AN	0.1424	0.409
AP	0.4919	0.021^*c*^
AK	0.3499	0.061
TN	0.5644	0.002^*b*^
SOM	0.4443	0.038^*c*^
2020		
pH	0.1433	0.404
AN	0.1972	0.253
AP	0.6252	0.002^*b*^
AK	0.3602	0.06
TN	0.0871	0.61
SOM	0.0482	0.792

a Soil physicochemical properties: TN, total nitrogen; SOM, soil organic matter; AN, available nitrogen; AP, available phosphorus; AK, available potassium.

b Significant differences at *P *< 0.01.

c Significant differences at *P *< 0.05.

## DISCUSSION

### Crop yield and soil fertility in different rotation-fallow planting systems.

Our results showed that treatment D significantly increased paddy yields in 2019 and 2020 than in 2018 (Fig. 1), which is consistent with Singh et al. (22). This might be attributed to the fact that paddy-upland crop rotation systems could improve soil fertility, crop diversity, and crop yields (23). Moreover, the soil available nutrients, AN, AP, and AK, of treatment D were indeed higher than those of other treatments, indicating that the frequent alternating cycles of anaerobic and aerobic conditions in the paddy-upland rotation system promoted carbon and nitrogen cycles, thereby improving the content of available N, P, and K in the soil (24, 25). The lowest yield was obtained in the “milk vetch - spring soybean - autumn soybean” rotation treatment, because the treatment employed the least amount of fertilizer for nourishing the land throughout the year (Table S1). Furthermore, Kirchhof et al. (26) reported that legumes can serve as land-farming crops requiring minimal fertilizer application and management practices, thereby presenting low yields. As a result of the long-term flood-induced barrier, treatment A presented with/had a low yield of “milk vetch - double cropping rice.” We found that the fast-acting nutrients, AN, AP, and AK, were lower in treatment A than in the other treatments in the second year of continuous cropping, which is consistent with the results of Zhang et al. (27) and Qin et al. (28). However, the pH and SOM of soil physicochemical properties showed a continuous decreasing trend in different rotational fallow cropping systems, which may be attributed to the constant transition of flooded-aerobic-flooded soil environmental condition, and thus, producing reciprocal barriers to soil fertility, as was reported by Witt et al. (29).

### Soil bacterial community structure in different rotation-fallow planting systems.

The results of our study showed that the rice field rotation-fallow pattern had a significant effect on soil bacterial community diversity, with phyla abundance and diversity increasing with the number of years of cultivation (Table 2). Among them, the bacterial α-diversity index of continuous cropping in treatment A planted with double-season rice in different crop rotation-fallow systems in 2018 was higher than in the other treatments; however, the diversity of soil bacterial phyla in each treatment significantly increased in 2020, with no considerable variation among treatments. The reason for this may be attributed to high soil water content of planted rice, which promotes the activities of anaerobic microorganisms and the relative abundance of anaerobic bacteria, phylum *Nitrosospirillum*, in continuous rice cropping in treatment A more than in other treatments. Furthermore, the study showed that the anaerobic bacteria, phylum *Nitrosospirillum* prefers aquatic environment (30). In addition, the relative abundance of anaerobic *Acidobacteriota* and *Chloroflexi*, which were the dominant phyla in treatment A, were considered higher compared to the other treatments. He et al. (31) demonstrated that rice soils have high nitrogen cycle microbial diversity index than other rotational soils, which is might be attributed to the high-water content of rice soils. The significant effect on soil bacterial community structure during the period of rice field crop rotation-fallow pattern may be attributed to the inconsistent soil environment factor required by bacteria in paddy and dry land. In addition, another reason may be due to the diversity of plants in crop rotation-fallow ecosystem, resulting in an increase in root secretions, which affects the community structure of soil microorganisms (32). The bacterial community structure of different crop rotation-fallow systems significantly differed the most in 2019. The results suggest that the rotation provides greater concentration and diversity of organic matter, both of which may lead to significant differences in microbial community structure and diversity among different crop rotation fallow systems. Xuan et al. (16) found no relationship between high rice yield and high soil bacterial diversity; however, in our study design, we found a positive relationship between soil bacterial phyla diversity and crop yield. In 2020, the anaerobic environment of the rice soil habitat was constructed under flooding conditions due to the increased water content, which resulted in an increase of bacterial diversity index. However, this condition decreased the dominant phylum relative abundance (Table S3) due to the preference of the phylum Aspergillus for an aerobic environment (33).

### Key factors influencing soil bacterial community structure under different rotation-fallow planting systems.

According to redundancy analysis, AP was the key influencing factor on soil bacterial community structure under different rotation-fallow tillage systems in 2019 and 2020. However, our result is different from that of other studies, which have reported that pH, SOM, soil water content, and soil temperature are the key factors affecting soil microbial community structure in the crop rotation-fallow model (34, 35). We speculate that this result may be attributed to the higher number of phosphorus solubilizing bacteria. Yaghoubi et al. (36) reported the presence of phosphorus solubilizing promoters in interroot soils of cotton, rice, maize, wheat, and sugarcane crops. Moreover, we found that most of bacteria in our study were identified as *Bacillus* spp. and Pseudomonas spp. microorganisms, which promote the reactivity of phosphorus by producing inorganic and organic acids, phosphatases, iron carriers, and chelating compounds (37). We also found that the soil bacterial community structure of the phyla *Firmicutes*, *Acidobacteria*, and *Bacteroidetes* in the experiment were mostly bacilli bacteria, whose abundance was improved by the effectiveness of AP (33). Therefore, AP might be the key determinant for the limited bacterial community structures in the different rotation-fallow systems following a 3-year field experiment.

### Conclusions.

Different agricultural management approaches can influence soil bacterial community structure, which results in significant change in soil property and productivity. In this study, different crop rotation-fallow systems increased the diversity of soil bacterial communities but decreased phyla relative abundance as the number of years of crop rotation increased. In addition, although there were several influencing factors for the soil bacteria community structure in 2018, 2019, and 2020, AP might be the key factor for the limited bacteria community structure in various rotation-fallow systems following a 3-year experiment. Moreover, the AP content significantly increased and stabilized the relative abundance of phosphorus-promoting bacteria, thereby facilitating the utilization of phosphorus fertilizers. Our results indicate that different crop rotation-fallow systems influence the intrinsic relationship between bacterial community structure and soil physicochemical properties.

## MATERIALS AND METHODS

### Study area and crop rotation experiment.

**FIG 5:**
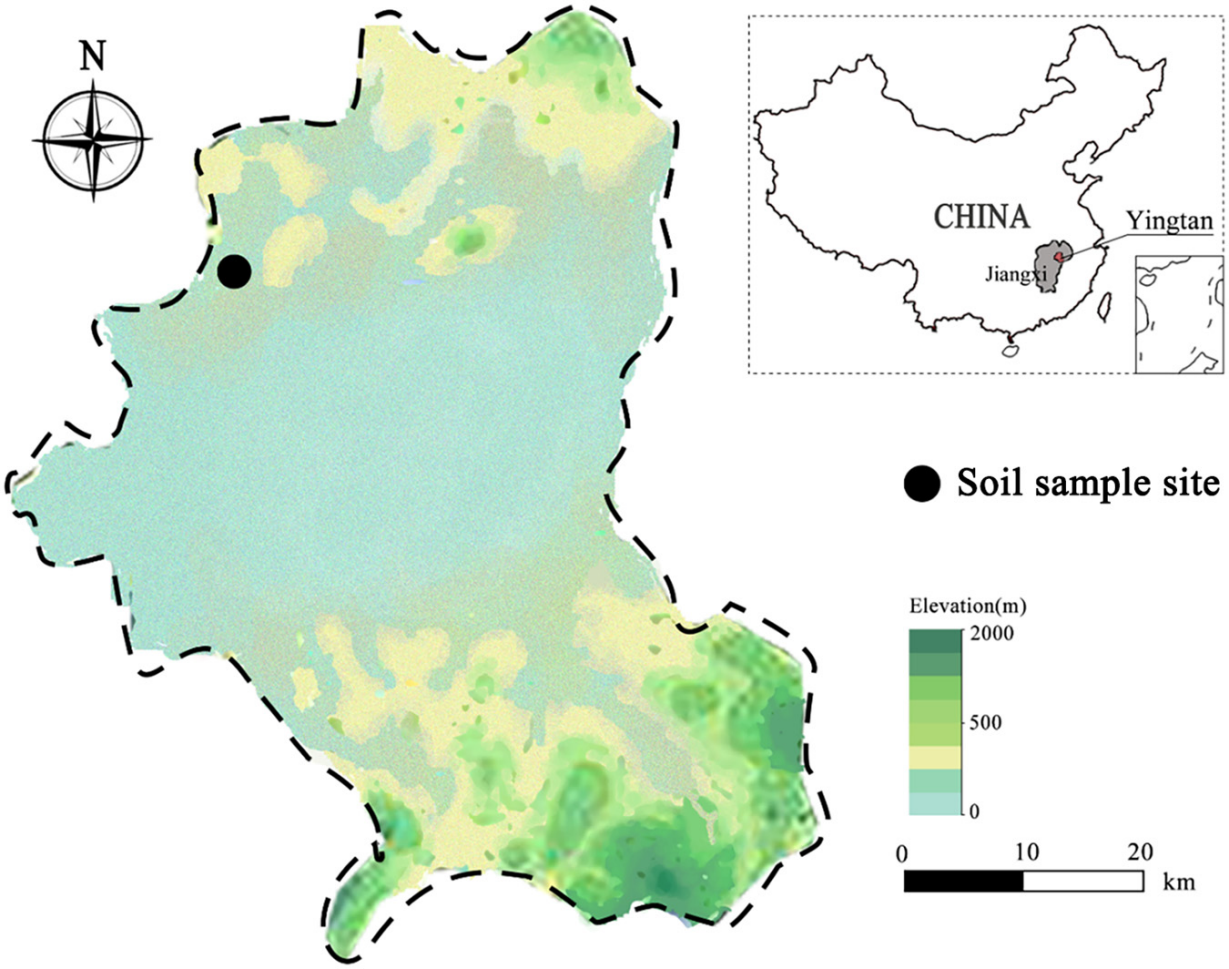
Schematic map showing the experimental layout among different rotational cropping systems.

The study site (Fig. 5) is located in Yingtan City, Jiangxi Province, China (28°14′ 8″ N, 116°51′ 22″ E), and the cropping history of this region revealed that monoculture rice cultivation was the conventional practice. The study site is situated at the subtropical climate, with an annual average temperature and rainfall of 17.5°C and 1,796.8 mm, respectively. The pretest soil parameters were evaluated as follows: 1.79 g·kg^−1^ of total nitrogen, 30.07 g·kg^−1^ of organic matter, 162.67 mg·kg^−1^ of available nitrogen, 86.1 mg·kg^−1^ of available phosphorus, 129.67 g·kg^−1^ of available potassium, and pH 5.07. We collected soil samples from block design experiments of five cropping systems (area of each = 66.7 m^2^) with three replicates. From 2018 to 2020, A (CK), BCD, and E were designed for continuous, rotation, and annual fallow cropping systems, respectively (Table 4). The crop varieties that were grown in the 3-year experiment are as follows: milk vetch (*Astragalus sinicus L.*), oilseed rape (*Brassica napus L.*), early rice (Oryza sativa *L.*), late rice (Oryza sativa *L*), soybean (Glycine max (*L.*) *Merrill*), maize (Zea mays *L.*), sugarcane (*Saccharum officinarum L.*), and sweet potato [*lpomoea batatas* (*L.*) *Lam.*]. The sowing volume/density and period, as well as the harvesting period for each crop treatment are as follows: (i) the average annual sowing volume of early rice was ~45 kg·hm^−2^, with the transplantation and harvesting performed in April and July, consecutively. (ii) The average annual sowing volume of late rice was approximately 42 kg·hm^−2^, with the transplantation and harvesting performed in July and November, consecutively. (iii) The average annual sowing volume and cultivation period of purple yew was approximately 22.5 kg·hm^−2^, and in October, and the transplantation was performed in the following April during its blooming season. (iv) The average annual sowing density of oilseed rape was approximately 111,000 plants·hm^−2^, with the transplantation and harvesting performed in December and May, consecutively. (v) The average annual sowing density of soybeans was approximately 67,000 plants·hm^−2^, with the transplantation and harvesting of spring soybeans performed in April and June, while that of the fall soybeans performed in June and October, consecutively. (vi) The average annual sowing density of maize was approximately 67,000 plants·hm^−2^, with the sowing and harvesting performed in July and October, consecutively. (vii) The average annual sowing density of sugarcane was approximately 8,230 plants·hm^−2^, with the transplantation and harvesting performed in May and December, consecutively. (viii) The average annual sowing density of sweet potato was approximately 56,000 plants·hm^−2^, with the transplantation and harvesting performed in July and October, consecutively. The amount of fertilizer used for the five-crop rotation-fallow systems in 2018, 2019, and 2020 is shown in Table S1.

**TABLE 4:** Experimental design among different rotational cropping systems^*a*^

Treatments	2018	2019	2020	Cropping pattern
A (CK)	Milk vetch - double cropping rice	Milk vetch - double cropping rice	Milk vetch - double cropping rice	Continuous cropping
B	Milk vetch - spring soybeans - autumn soybeans	rape - sugarcane ‖ spring soybeans	Milk vetch - double cropping rice	Rotation
C	Milk vetch - early rice - maize ‖ sweet potato	Milk vetch - spring soybeans - autumn soybeans	Milk vetch - double cropping rice	Rotation
D	Rape - sugarcane ‖ spring soybeans	Milk vetch - early rice-maize ‖ sweet potato	Milk vetch - double cropping rice	Rotation
E	Fallow	Fallow	Milk vetch - double cropping rice	Fallow

a “-”: Multiple cropping; “‖”: intercropping.

### Sample collection.

Three sampling sites (14.9 m × 7.33 m) for each cropping systems were selected. Samples were randomly collected from five soil columns (0 to 20 cm) at each site from October 2018 to 2020. Following root-residue removal, the resulting highly homogenous soil samples were divided into two portions and treated as follows: one portion (~200 g) was packed in a sterile plastic bag and transported to the laboratory on ice and stored at −80°C prior to molecular analysis. The remaining ~1 kg was air dried for physicochemical property assessment.

### Soil physicochemical properties.

Total nitrogen (TN) was determined via Automatic Flow Injection following digestion in H_2_SO_4_. Soil organic carbon (SOM) was determined via K_2_Cr_2_O_7_ oxidation. Soil pH was determined at a soil:water ratio of 1:2.5. Soil moisture content was measured by comparison of fresh and dried (105°C; 24 h) weights of samples (Lu, 2000). Alkali-hydrolyzable nitrogen (AN) was measured using the alkali diffusion method. Available phosphorus (AP) was determined following extraction with 0.5 M NaHCO_3_, and lastly, available potassium (AK) was determined via atomic absorption spectroscopy (38).

### DNA extraction, amplification, and sequencing.

Total soil DNA was extracted from 0.5 g of freeze-dried soil using the FastDNA SPIN Kit for soil samples (MP Biomedicals, Santa Ana, CA, USA). The primer of 338F (5′-ACTCCTACGGGAGGCAGCAG-3′)/806R (5′-GGACTACHVGGGTWTCTAAT-3′) was used to amplify the 16S rRNA V3-V4 region of total bacteria (39). All the samples were amplified in triplicate. The 16S rRNA gene was amplified with the forward primers tagged with a 5′-nucleotide barcode. The purified PCR products were sequenced on the Illumina MiSeq (300-bp paired-end reads) platform (Illumina Inc., San Diego, CA, USA) at the Shanghai Majorbio Bio-pharm Technology Co., Ltd., Shanghai, China.

### Statistical analyses.

The quality of the downstream analysis was evaluated by filtering raw pair-end reads for the different samples, according to exact matching of barcodes (39). Reads presenting one or more mismatches with the barcode sequences or at least two mismatches with primer sequences were discarded (40). The resulting reads were merged using FLASH (Fast Length Adjustment of Short reads) of at least 10-bp overlapping regions (34), and sequences exhibiting lengths beyond the expected range (200 to 500 bp), or those containing any ambiguous bases 41, were discarded as wel. Finally, we obtained the pure reads of 16S rRNA gene in 2018, 2019, and 2020 as 1,381,759, 1,256,509, and 717,018, respectively. The default parameters were used to remove chimeras by the Ribosomal Database Project (RDP) FunGene pipeline (42). Following filtration and chimera removal, *de novo* operational taxonomic unit (OTU) selection was performed using the UCLUST algorithm at 97% sequence identity. Chimeric sequences, chloroplast, and mitochondrial OTUs, as well as singleton OTUs were discarded from the final OTU table. Finally, 2,997, 4,706, and 6,303 OTUs were obtained for the 16S rRNA genes in 2018, 2019, and 2020, respectively. The α- and β-diversities of the 16S rRNA were evaluated via QIIME software (Version 1.9.0) and unweighted UniFrac distances, respectively (43).

Soil physicochemical properties and bacterial gene abundance were analyzed via SPSS software (v.18.0, Chicago, IL, USA) and one-way ANOVA, respectively, and Duncan was tested at a significance level of *P < *0.05. Variations in bacterial community structures between soil samples were determined via nonmmetric multidimensional scaling [NMDS] (44). Differentiating of the bacterial community structures was performed using ANOSIM (45). The composition of 16S rRNA genes in different succession stages was depicted via Community barplot analysis diagram (46). The environmental variables of community composition ranking were analyzed via Bray-Curtis distance redundancy analysis in Canoco 5.0 (Microcoputer Power, Ithaca, NY, USA). The impact of each environmental variable toward the description of variation in bacterial community structure was determined by forward selection (47).

### Data availability.

All 16S rRNA gene sequences obtained in this study have been deposited in the GenBank Sequence Read Archive (SRA) under accession numbers PRJNA758723, PRJNA660916, and PRJNA758730.
