# Supplementary material for: Impacts of Rotation-Fallow Practices on Bacterial Community Structure in Paddy Fields
Source: Microbiol Spectr. 2022 Jul 27;10(4):e00227-22. doi: 10.1128/spectrum.00227-22 (PMC9431176; doi:10.1128/spectrum.00227-22)
Supplement: Supplemental file 1 — Supplemental material. Download spectrum.00227-22-s0001.pdf, PDF file, 0.2 MB [file spectrum.00227-22-s0001.pdf]

TS 1. Amount of fertilizer applied of different rotational cropping systems

	Treatments	Apply fertilizer ($kg·hm^{-2}$)	Total amount of fertilizer ($kg·hm^{-2}$)
2018	A1*	N: 343; P: 136; K: 136	N: 1371.25; P: 440.75; K: 440.75; Ca-Mg-P: 899
	B1	N: 92	
	C1	N: 182.75; P: 79.25; K: 79.25	
	D1	N: 753.5; P: 225.5; K: 225.5; Ca-Mg-P: 899	
	E1	0	
2019	A2	N: 343; P: 136; K: 136	N: 1371.25; P: 440.75; K: 440.75; Ca-Mg-P: 899
	B2	N: 753.5; P: 225.5; K: 225.5; Ca-Mg-P: 899	
	C2	N: 92	
	D2	N: 182.75; P: 79.25; K: 79.25	
	E2	0	
2020	A3	N: 343; P: 136; K: 136	N: 1715; P: 680; K:680
	B3	N: 343; P: 136; K: 136	
	C3	N: 343; P: 136; K: 136	
	D3	N: 343; P: 136; K: 136	
	E3	N: 343; P: 136; K: 136	

* Treatments: A: milk vetch - double cropping rice → milk vetch - double cropping rice → milk vetch - double cropping rice; B: milk vetch - spring soybeans - autumn soybeans → rape - sugarcane | | spring soybeans → milk vetch - double cropping rice; C: milk vetch - early rice - maize | | sweet potato → milk vetch-spring soybeans - autumn soybeans → milk vetch - double cropping rice; D: rape - sugarcane | | spring soybeans → milk vetch - early rice - maize | | sweet potato → milk vetch - double cropping rice; E: fallow → fallow → milk vetch - double cropping rice.

TS 2. Results of ANOSIM (analysis of similarities) test performed based on Bray-Curits distance for bacterial community structure.

	R	*P* value
2018 DE*	-0.0741	0.587
2020 BCDE	-0.0123	0.519

* Treatments: A: milk vetch - double cropping rice → milk vetch - double cropping rice → milk vetch - double cropping rice; B: milk vetch - spring soybeans - autumn soybeans → rape - sugarcane || spring soybeans → milk vetch - double cropping rice; C: milk vetch - early rice - maize || sweet potato → milk vetch-spring soybeans - autumn soybeans → milk vetch - double cropping rice; D: rape - sugarcane || spring soybeans → milk vetch - early rice - maize || sweet potato → milk vetch - double cropping rice; E: fallow → fallow → milk vetch - double cropping rice.

**represent significant differences at p<0.01.

TS 3. Phylum level community structure of soil bacteria under different rotation-patterns

	Treatments	*Proteobacteria*	*Acidobacteriota*	*Chloroflexi*	*Actinobacteriota*	*Firmicutes*	*WPS-2*	*unclassified_k__norank_d__Bacteria*	*Nitrospirota*	*Verrucomicrobiota*	*Myxococcota*	*Bacteroidota*	*Patescibacteria*	*Desulfobacterota*	*Planctomycetota*	*Gemmatimonadota*	*RCP2-54*	*Methylomirabilota*	*Cyanobacteria*	*MBNT15*	*Sva0485*	*others*
	A1*	15990.67 ± 1382.41Aa	15546.67 ± 81.00Aab	10818.33 ± 2208.27Aab	2480.33 ± 256.70Ad	897.33 ± 187.18Bd	740.33 ± 31.66Ab	532.67 ± 165.98Ba	4091.00 ± 233.08Aa	2654.67 ± 499.76Aab	1206.00 ± 43.35Aa	1791.00 ± 59.41Ab	1299.00 ± 51.45Ab	1835.00 ± 162.92Aa	1500.67 ± 450.68Aa	647.33 ± 47.06Bbc	392.67 ± 31.66Bb	585.00 ± 162.89Aa	431.33 ± 129.03Aa	637.00 ± 14.80ABa	207.00 ± 36.51Ba	1810.67 ± 259.33Aa
	B1	21019.00 ± 5576.72Aa	21518.00 ± 8260.11Aa	8519.00 ± 1843.42Aab	4108.67 ± 565.44Bc	1444.33 ± 397.18Acd	2155.00 ± 1296.94ABab	553.67 ± 116.93Aa	1824.00 ± 1040.49Ab	3655.67 ± 1379.00Aa	1016.00 ± 348.77Aa	1642.33 ± 499.56Ab	1653.00 ± 220.25ABab	658.67 ± 375.45Abc	2138.67 ± 790.43Aa	950.00 ± 220.52Aab	784.00 ± 181.02Aa	398.67 ± 245.83Aab	344.67 ± 165.72Aa	409.33 ± 293.99Aab	95.00 ± 50.08Bbc	2127.33 ± 208.14Aa
2018	C1	31522.00 ± 17061.13Aa	10067.00 ± 4321.56Bb	12971.00 ± 5298.43Aa	8255.00 ± 806.57Aa	3207.33 ± 78.64Aab	3297.67 ± 2007.56Aa	611.67 ± 364.36Ba	514.00 ± 130.15Bc	1668.67 ± 904.99Ab	395.00 ± 33.15Bb	1229.00 ± 335.04Bb	2813.00 ± 1237.69Aa	278.67 ± 149.38Bc	1608.33 ± 1362.65Aa	485.00 ± 124.37Bc	374.67 ± 54.63Bb	74.33 ± 13.28Bb	256.67 ± 161.07Aa	102.00 ± 33.29Bb	42.00 ± 17.59Bc	1200.33 ± 122.85Ab
	D1	27165.00 ± 5047.95Aa	12838.33 ± 1327.87ABab	6972.67 ± 669.53Ab	5759.67 ± 1061.77Ab	3934.67 ± 1037.91Aa	1708.33 ± 256.66Bab	302.33 ± 80.53Ba	937.33 ± 243.94Abc	2500.67 ± 142.03Aab	1117.67 ± 135.82Aa	2768.00 ± 901.22Aa	2566.67 ± 311.70Aa	583.67 ± 251.45Abc	1599.00 ± 165.00Aa	1316.67 ± 218.18Aa	393.67 ± 111.39Bb	108.67 ± 9.87Bb	368.33 ± 81.13Aa	200.00 ± 70.08ABb	71.67 ± 31.53Ac	1885.33 ± 275.12Aa
	E1	22514.00 ± 6489.15Aa	17936.33 ± 5616.07Aab	7529.33 ± 590.34Ab	4143.40 ± 984.24Bc	2313.33 ± 1299.62Abc	2353.33 ± 500.35Aab	495.33 ± 106.51Ba	1408.00 ± 674.38Abc	3128.67 ± 343.42Aab	1042.00 ± 219.36Aa	1708.67 ± 244.38Ab	1663.67 ± 445.00Aab	953.00 ± 193.83Ab	2080.33 ± 478.55Aa	957.67 ± 315.38Aab	536.00 ± 220.40Bab	329.00 ± 287.36Aab	525.00 ± 264.14Aa	383.00 ± 208.76ABab	150.33 ± 49.52Aab	2063.33 ± 490.94Aa
	A2	12171.33 ± 2392.62Bb	14471.00 ± 2168.74Ab	6341.33 ± 1328.38Ba	2557.33 ± 599.00Ad	860.67 ± 38.28Bb	640.67 ± 164.88Ac	6443.00 ± 1842.99Aa	3985.00 ± 356.25Aa	806.33 ± 508.66Ba	1634.67 ± 226.40Aa	1287.33 ± 130.77Aa	295.67 ± 51.86Cb	1745.33 ± 388.08Aa	43.00 ± 8.54Bb	955.33 ± 118.70Aa	737.67 ± 121.82Ab	674.33 ± 125.02Aa	166.67 ± 37.45Bc	981.67 ± 310.88Aa	707.67 ± 79.94Aa	1123.00 ± 262.44Bab
	B2	23632.33 ± 7201.78Aa	8486.00 ± 957.90Bc	12469.00 ± 6762.35Aa	5733.67 ± 805.9Aab	2950.00 ± 1165.06Aa	5303.67 ± 2429.59Aa	1818.67 ± 1631.48Ab	1298.67 ± 520.50Ab	416.00 ± 130.64Ba	791.00 ± 112.30Ac	1378.67 ± 98.82Aa	3114.33 ± 1746.01Aa	773.33 ± 302.66Ab	78.33 ± 45.62Bab	634.67 ± 116.66Bb	525.67 ± 126.12Ab	291.00 ± 73.43Ab	305.00 ± 30.81Aa	310.33 ± 39.43Ab	262.00 ± 75.90Ab	741.33 ± 148.17Cb
2019	C2	17508.00 ± 1862.62ABab	25331.00 ± 1783.59Aa	9071.67 ± 2448.58Aa	5030.00 ± 776.96Bbc	1921.67 ± 1227.37ABab	2003.67 ± 624.87Abc	3266.67 ± 491.61Ab	973.00 ± 135.24Ab	1239.67 ± 409.34Aa	1086.33 ± 132.50Ab	769.67 ± 66.46Ab	446.00 ± 104.01Bb	597.33 ± 16.26Ab	102.67 ± 16.56Aab	869.67 ± 68.50Aa	3281.33 ± 1846.43Aa	381.33 ± 8.08Ab	209.33 ± 33.47Abc	448.67 ± 27.54Ab	167.67 ± 30.66Ab	1145.67 ± 226.86Aab
	D2	15095.67 ± 923.43Bb	14561.67 ± 3428.65Ab	9450.33 ± 2415.17Aa	6635.67 ± 790.51Aa	2005.33 ± 344.56Bab	4094.00 ± 503.11Aab	3052.33 ± 522.24Ab	801.00 ± 260.65Ab	410.67 ± 174.04Ba	861.67 ± 80.41Abc	873.00 ± 73.01Ab	1864.33 ± 602.38Aab	519.33 ± 200.00Ab	88.33 ± 21.96Cab	910.67 ± 70.44Ba	901.67 ± 157.38Ab	363.67 ± 105.31Ab	271.00 ± 58.23Aab	321.00 ± 98.32Ab	152.67 ± 72.95Ab	858.00 ± 191.25Cb
	E2	16842.00 ± 2417.89ABab	23141.00 ± 3271.33Aa	7197.00 ± 1287.76Aa	4367.00 ± 1543.53Bc	1869.00 ± 977.39Aab	1310.33 ± 581.60ABc	2685.33 ± 199.72Ab	1169.67 ± 1254.17Ab	1112.67 ± 714.45Ba	1481.00 ± 69.20Aa	917.67 ± 297.46Ab	414.00 ± 212.61Bb	834.67 ± 168.35Ab	119.67 ± 47.75Ca	809.00 ± 34.70Aa	1246.33 ± 175.37Ab	725.67 ± 145.51Aa	231.67 ± 62.01Aab	592.67 ± 78.13Ab	214.67 ± 38.44Ab	1354.33 ± 305.03Ba
	A3	8440.00 ± 1258.46Ca	9036.67 ± 766.38Ba	12786.33 ± 1833.38Aa	5362.67 ± 77.31Ba	1325.67 ± 3203.32Ab	222.67 ± 52.16Bb	313.00 ± 30.51Ba	1222.67 ± 178.06Ba	703.33 ± 177.35Ba	1548.33 ± 306.77Aa	998.33 ± 125.83Aa	540.00 ± 44.43Ba	1002.33 ± 192.42Ba	1226.33 ± 256.51Aa	615.00 ± 41.51Ba	83.00 ± 27.40Ca	653.00 ± 50.03Aa	433.00 ± 24.64Aa	382.67 ± 106.61Ba	199.67 ± 53.14Ba	1480.00 ± 157.04ABa
	B3	7406.00 ± 1155.74Ba	8423.00 ± 805.31Ba	11218.67 ± 1043.36Aab	6377.33 ± 922.55Aa	1710.33 ± 444.70Aab	682.00 ± 135.90Bab	272.00 ± 80.90Aa	463.33 ± 122.40Bb	633.67 ± 186.32Ba	1121.67 ± 22.05Aa	718.67 ± 57.88Aab	477.67 ± 77.05Bab	772.67 ± 3175.74Aa	834.00 ± 78.46Ba	602.00 ± 67.82Ba	103.00 ± 42.67Ba	366.33 ± 110.09Ab	469.00 ± 269.70Aa	161.00 ± 21.07Ab	57.67 ± 6.11Bb	1378.67 ± 103.39Ba
2020	C3	6575.00 ± 448.01Ca	9620.67 ± 2831.58Ba	8935.00 ± 3032.89Aab	5271.00 ± 130.55Ba	1273.00 ± 229.14Cb	825.67 ± 388.57Aa	307.67 ± 102.48Ba	466.00 ± 130.61Bb	733.33 ± 379.91Aa	1002.33 ± 39.80Aa	607.67 ± 78.78Ac	367.00 ± 85.86Bb	704.67 ± 194.95Aa	815.00 ± 314.02Aa	600.00 ± 52.05Ba	146.33 ± 37.74Ba	426.33 ± 189.69Ab	320.33 ± 199.92Aa	115.67 ± 39.88Bb	47.00 ± 39.40Bb	1271.67 ± 153.28Aa
	D3	8795.00 ± 2360.60Ba	7360.00 ± 1940.09Ca	10169.33 ± 2492.29Aab	7435.33 ± 2090.36Aa	2196.67 ± 526.25Ba	697.67 ± 248.55Cab	232.33 ± 15.95Ba	572.33 ± 337.59Ab	703.00 ± 321.14Ba	1327.67 ± 510.50Aa	900.00 ± 234.09Aab	511.00 ± 102.24Bab	965.33 ± 415.87Aa	1034.67 ± 375.30Ba	575.67 ± 118.84Ca	102.00 ± 15.52Ca	274.67 ± 115.49ABb	329.00 ± 188.46Aa	111.00 ± 54.75Bb	51.67 ± 43.94Ab	1421.67 ± 139.93Ba
	E3	9352.33 ± 3086.46Ca	23141.00 ± 1394.72Ba	8215.00 ± 1366.68Ab	7489.33 ± 1266.49Aa	1541.33 ± 403.00Aab	431.00 ± 264.92Bab	253.67 ± 55.77Ba	795.00 ± 470.58Aab	467.33 ± 265.90Ba	1548.00 ± 410.92Aa	830.33 ± 295.23Aab	371.67 ± 90.80Bb	981.67 ± 210.44Aa	736.00 ± 205.92Ba	750.33 ± 304.07Aa	104.33 ± 32.86Ca	386.67 ± 115.49Ab	524.33 ± 475.67Aa	203.33 ± 114.29Bb	102.33 ± 87.27Ab	1250.33 ± 107.01Ba

* Treatments: A: milk vetch - double cropping rice → milk vetch - double cropping rice → milk vetch - double cropping rice; B: milk vetch - spring soybeans - autumn soybeans → rape - sugarcane || spring soybeans → milk vetch - double cropping rice; C: milk vetch - early rice - maize || sweet potato → milk vetch-spring soybeans - autumn soybeans → milk vetch - double cropping rice; D: rape - sugarcane || spring soybeans → milk vetch - early rice - maize || sweet potato → milk vetch - double cropping rice; E: fallow → fallow → milk vetch - double cropping rice.

TS 4. Results of ANOSIM (analysis of similarities) test performed based on Bray-Curits distance for bacterial community structure.

	R	*P* value
2018	0.5244	0.001**
2019	0.8252	0.001**
2020	0.4415	0.004**

**represent significant differences at $P<0.01$.
